# Allelic Variation and Selection in Effector Genes of *Phytophthora infestans* (Mont.) de Bary

**DOI:** 10.3390/pathogens9070551

**Published:** 2020-07-09

**Authors:** Juan G. Morales, Astrid E. Gaviria, Elizabeth Gilchrist

**Affiliations:** 1 Group and Laboratory of Fitotecnia Tropical, Departamento de Ciencias Agronómicas, Facultad de Ciencias Agrarias, Universidad Nacional de Colombia sede Medellín, Medellín, 050034 Antioquia, Colombia; aegaviriag@unal.edu.co (A.E.G.); egilchri@eafit.edu.co (E.G.); 2 Universidad EAFIT, 050034 Medellín, Colombia

**Keywords:** *Phytophthora infestans* sensu lato, *Solanum* spp., wild hosts, evolutive selection, effector proteins

## Abstract

*Phytophthora infestans* is a devastating plant pathogen in several crops such as potato (*Solanum tuberosum*), tomato (*Solanum lycopersicum*) and Andean fruits such as tree tomato (*Solanum betaceum*), lulo (*Solanum quitoense*), uchuva (*Physalis peruviana*) and wild species in the genus *Solanum* sp. Despite intense research performed around the world, *P. infestans* populations from Colombia, South America, are poorly understood. Of particular importance is knowledge about pathogen effector proteins, which are responsible for virulence. The present work was performed with the objective to analyze gene sequences coding for effector proteins of *P. infestans* from isolates collected from different hosts and geographical regions. Several genetic parameters, phylogenetic analyses and neutrality tests for non-synonymous and synonymous substitutions were calculated. Non-synonymous substitutions were identified for all genes that exhibited polymorphisms at the DNA level. Significant negative selection values were found for two genes (*PITG_08994* and *PITG_12737*) suggesting active coevolution with the corresponding host resistance proteins. Implications for pathogen virulence mechanisms and disease management are discussed.

## 1. Introduction

Plant pathogens secrete molecules whose function is to facilitate host localization, adherence, penetration, colonization, nutrient uptake and reproduction into host tissues [1]. As a result, disease is induced causing tissue damage that may end in plant death. A group of such molecules, collectively named effector proteins, is dedicated to promote pathogenicity through manipulation of host metabolism or suppression of its immune system [2]. As a counterpart, host plants have developed a complex immune system to detect pathogen signals and trigger diverse pathways of defense responses [3,4,5]. When a plant defense protein, named an R protein, recognizes an effector protein from the pathogen, the hypersensitive response is activated, and this effector is named thereafter as an avirulence protein. The hypersensitive response is a typical response in the interaction of different physiological races of the oomycete pathogen *Phytophthora infestans* (Mont.) de Bary with potato differentials expressing corresponding R proteins [3,4,6].

Plant *R* and pathogen effector genes have evolved in an arms race-like system of attack and counterattack [6]. Therefore, evolutionary selection pressure is exerted over the pathogen effector domains and on corresponding plant R resistance proteins. Pathogens can lose, suppress gene expression or change the avirulence gene sequence to avoid recognition by the plant defense system, and in turn, plants can evolve a novel resistance gene to recognize effector proteins. This co-evolutionary process creates polymorphisms in corresponding gene sequences in both the host and the pathogen that leave a footprint in the genome sequences. Allelic variation in R and effector genes seen as a result of this arms-race has been described in several pathosystems including oomycetes and their plant hosts [2,7,8,9].

Oomycetes are a group of microorganisms in which devastating plant pathogens are classified, mainly in the genus *Phytophthora*. Oomycete effectors such as *scr74* (74 amino acid secreted cysteine-rich protein) from *Phytophthora infestans* exhibit extreme levels of amino acid polymorphisms and evidence of diversifying selection and recombination [10]. Extreme polymorphisms have been observed in the *Arabidopsis thaliana* RPP13 (Resistance to *Peronospora parasitica* 13) R protein and its corresponding ATR13 (*Arabidopsis thaliana* Recognized 13) avirulence determinant from the oomycete pathogen *Hyaloperonospora parasitica* (current name *H. arabidopsidis*) [11]. In the *ATR1^NdWsB^* gene from *H. parasitica* and the cognate *RPP1* gene from *A. thaliana*, 90 non-synonymous and only 9 synonymous segregating polymorphisms were found in six different alleles sequenced [12]. In addition, positive selection of the C terminal part of the RxLR effector proteins has been found at least for two-thirds of the evaluated paralogues of RxLR gene families from *P. sojae*, *P. ramorum* and *H. parasitica* [13]. Even more, evidence suggesting functional redundancy of the RxLR class of effector genes has been identified in *Phytophthora* sp. [6,14,15].

*Phytophthora infestans* like other oomycetes deploys effector proteins into host cells [14]. *Avr3a* was the first *P. infestans* RxLR avirulence gene cloned and sequenced [16]. Since then many other RxLR genes have been identified, cloned and sequenced [3,4,15,17,18]. In contrast to the highly divergent alleles of *ATR1* and *ATR13* from *H. parasitica*, *Avr3a* has two prevalent alleles: *Avr3aKI* (dominant avirulent form) and *Avr3aEM* (virulent form). Avr3aKI protein is recognized in potato plants containing its cognate R3a resistance protein, inducing the hypersensitive response. *Avr3a* was sequenced from 55 different isolates, and only these two alleles (*Avr3aKI* and *avr3aEM* changing amino acids S19C, E80K and M103I) showing 100% correlation with the virulence phenotype were found [16]. *Avr3a* also has two paralogues, *Pex147*-like pseudogenes, *Pex147-2* and *Pex147-3* [16]. Recent evidence suggests that *Avr3a* has experienced a selective sweep associated with negative selection [19].

Effector gene cloning and diversity studies have been useful to accelerate the discovery of corresponding resistance genes in the plant host and therefore plant breeding, as exemplified during the last years for the *P. infestans*–*Solanum* sp. pathosystem [20,21]. In this work, the main objective was to analyze the allelic variation in selected *P. infestans* RxLR effector genes from isolates collected in a diverse range of hosts and geographical locations in Colombia. Genes studied in the present research were selected from previously published investigations [15,17,18]. Non-synonymous substitutions and signatures of selection were identified suggesting active coevolution in the pathosystem.

## 2. Results

### 2.1. Phytophthora Infestans Isolates

Twenty-nine isolates were obtained from different hosts and regions in Colombia (Table 1). Remarkably, 3 isolates (M-2, M-3, M-10) were collected from 3 different wild *Solanum* sp., plant species classified within the Anarrhichomenum and Basarthrum complexes, that are known for their difficult identification and which are probably new hosts for *P. infestans* from Colombia; however, further confirmation is needed [22].

### 2.2. Mating Type

Crosses in Petri dishes did not show oospores, which are produced by sexual reproduction indicating only one mating type. In addition, PCR amplification using DNA from all isolates with primers S1A (5′-AGGATTTCAACAA) and S1B (5′-TGCTTCCTAAGG) produced a DNA fragment of about 1250 bp, which is the expected size of an amplification product linked to the S1 locus that has been associated with the A1 mating type. Results obtained suggest that isolates are of the A1 mating type of *P. infestans*.

### 2.3. Sequence Analysis of Effector Genes

Genes studied in the present research were selected from previously published investigations. The main characteristics for selecting effector genes were as follows: they were up-regulated during host infection, showed non-synonymous substitutions, exhibited unique single-nucleotide polymorphisms (SNPs) in the highly aggressive isolate 06_3928A, suppressed host immune responses or induced defense responses such as cell death in *P. infestans* hosts or non-hosts as *Capsicum* sp. Genes *PITG_17063*, *PITG_06099* and PITG_*12737* code for the RXLR class of effector proteins; gene *PITG_15980* encodes for a putative glycoside hydrolase protein; gene *PITG_23123* encodes for a small cysteine-rich protein SCR50; and gene *PITG_08944* encodes for a putative endoglucanase protein [15,17,18].

All polymorphic sites were considered for the analysis. As a result, no polymorphic sites were identified for gene *23123*. For all other genes, different numbers of polymorphic sites were identified for each gene ranging from 52 for gene *08944* to 3 for gene *15980*. The highest haplotype diversity was found for gene *06099* and the lowest for gene *08944* (Table 2).

All effector genes that showed polymorphisms at the DNA sequence level exhibited non-synonymous and synonymous amino acid substitutions in the corresponding putative protein sequence (Table 3 and Supplementary Figures S1–S5).

For putative protein 06099, five non-synonymous substitutions located in four polymorphic sites were identified (Q39P; S63R; E80Q; E80K; K153R); for putative protein 08944, 27 non-synonymous polymorphic sites were found (L11S; G160C; V173X; X182C; E185Q; V190I; G192D; P202S; gap203E; H206Y; V207D; A208V; P209S; D210Y; I212P; G217T; R219W; H220S; G221P; A222T; F223I; P224L; W228R; gap229R; D230H; D231K; C232F); ten non-synonymous amino acid substitutions were found for putative protein 12737 (R57E; R77I; V80L; V94G; G95C; G95V; M99L; Y121F; E127Q; G147V; D150N); four non-synonymous substitutions in three polymorphic sites were identified for putative protein 17063 (I9P; I9S; S10P; Q99L) and two non-synonymous substitutions were found for putative protein 15980 (F303Y; G309R) (Table 4). Some polymorphic sites were located within the signal peptide region of the putative proteins (08944 L11S; 17063 I9P; I9S; S10P). One polymorphism in putative protein 12737 (R57E) was located within the RxLR motif, which is essential for protein translocation to the host cell cytoplasm (Table 4) [16]. Remaining non-synonymous substitutions were localized in the functional C-terminal portion of the protein, which has been identified as the region responsible for virulence (Supplementary Figures S1–S5) [13,24,25].

Neutrality tests showed significantly negative values for genes *08944* and *12737*, suggesting possible negative selection (Table 5). Some polymorphisms may change the physicochemical properties of the amino acid and, depending on several factors, potentially change the structure and functional properties of the corresponding protein [26,27]. This fact is particularly important when such substitutions are within the C terminal region of the effector proteins because this may induce changes in the function of virulence. In the present work, polymorphic substitutions were identified in which one amino acid was changed for another amino acid with a different charge, size or functional group, such as in gene *12737* (Y121F; E127Q; D150N) and gene *15980* (F303Y; G309R). Further research is needed to know if these substitutions have an effect on the virulence properties of corresponding protein.

Little diversity was found within the set of sequences of each gene. Similar results have been identified in genes coding for virulence and avirulence proteins of *P. infestans* in which one or few amino acid substitutions may be responsible for the change between virulence and avirulence phenotypes as observed for the *Avr3a* gene [16]. Phylogenetic analysis of DNA sequence alignments showed specific significant groups for each gene tested with a bootstrap value above 50% (Supplementary Figures S6–S10). In gene *06099*, a first significant group was identified for isolates Selva, UR9 and UR18, meanwhile isolates ST4 and MB1 were in a second group. Isolates OP2 and GA1 collected in the department of Huila, Colombia, together with isolate MB1 from Antioquia, showed a group in gene *12737*. In gene *15980*, isolates JA5, M7 and M-1 collected in Antioquia, Colombia, from *S. quitoense* (JA5 and M7) and *S. betaceum* (M-1) formed a group with the corresponding reference gene from isolate T-30. Sequences of gene *17063* formed two significant groups: in the first there were clustered isolates UR18 and JA4 collected in Antioquia from *S. quitoense*; in the second were isolates JA5 and GA1 collected in Antioquia and Huila, respectively, from *S. quitoense*. Isolates OP2 and JA6 collected from *S. quitoense* in Huila and Antioquia, respectively, formed a significant group for gene *08944*. No strong relationships were identified between sequence variants and the geographical or host origin of the isolate for any of the genes sequenced (Supplementary Figures S6–S10).

## 3. Discussion

Populations of the oomycete *Phytophthora infestans* sensu lato that cause the late blight disease have been the subject of intense studies worldwide because they cause large losses in potato and tomato crops. Potato and tomato plants are well known *P. infestans* hosts; however, this pathogen affects a number of Andean fruit crops such as lulo, tree tomato, uchuva, pear melon and wild *Solanaceous* species [22]. Despite a large investment in time and resources to study the *P. infestans*/*Solanum* spp. pathosystems, several aspects of its biology are still poorly understood mainly in South America [22]. In the present work, three isolates (M-2, M-3, M-10) were collected from wild *Solanum* sp. hosts, classified within the Anarrhichomenum and Basarthrum complexes, which are the subject of further identification. In the Andes range, new *Solanum* species are often discovered suggesting a large gap in the knowledge about this important genus. This is of particular importance for the Anarrhichomenum and Basarthrum complexes of *Solanum* sp. because plants classified in these groups are hosts of new variants of *P. infestans* that may play important, but mostly unknown, roles in late blight epidemics [22,28]. Even more unknown is the whole host range of *P. infestans* sensu lato, its ecological relationships and the extent to which these relationships influence epidemies in host crops. Gómez [28] recently identified one isolate from a commercial potato variety that significantly increased the parameters determinant of its biological fitness when it was inoculated on a wild host (*Solanum* sp.) and then was re-inoculated on the same potato host. These results suggest that wild hosts may have an important role on pathogen survival, fitness, diversity and other relevant factors for *P. infestans* sensu lato populations, which may be of crucial importance for disease dynamics on host crops. Therefore, it will be very important to intensify and broaden research on areas not previously explored for host identification and ecological interactions, information which may be useful for disease management [22]. In the present work, DNA or putative protein sequence variants observed in isolates M-2, M-3 and M-10 obtained from the Anarrhichomenum and Basarthrum complexes of *Solanum* sp. plants did not cluster in a particular group, indicating that they were not specific of isolates affecting those taxonomic complexes. Further research is required to investigate if isolates from wild hosts can cause disease in crop hosts and the implications they may pose in late blight management.

In Colombia, the *P. infestans* population has predominantly shown the A1 mating type. The A2 mating type was only reported in one isolate from uchuva (*Physalis peruviana*) [29]. Strikingly, later it was reported that uchuva exhibits histological responses typical of an incompatible interaction when infected with *P. infestans* [30]. No other work has reported the A2 mating type or *P. peruviana* as a host, suggesting that the A1 mating type predominance is still present as observed in the present work. Research about genetic diversity, coevolution, fungicide resistance, aggressiveness, host range, plant–pathogen interactions and other aspects are of key importance for designing better or novel management strategies for the late blight disease. Plant–pathogen coevolution at the molecular level has been investigated for a number of pathosystems including *P. infestans*/*Solanum* sp. Several plant resistance and pathogen effector genes have been cloned, sequenced and their expression during infection of the host analyzed. Plant–pathogen coevolution studies have generated useful information to accelerate plant breeding programs as exemplified by the ipiO effector gene from *P. infestans* [21,31,32].

In the present research, the sequence of six genes coding effector proteins from *P. infestans* sensu lato isolates collected from several hosts and different municipalities were analyzed. Genes *PITG_17063*, *PITG_06099* and *PITG_12737* code for the RXLR class of effector proteins; gene *PITG_15980* codes for a putative glycoside hydrolase protein; gene *PITG_23123* codes for a small cysteine-rich protein SCR50; and gene *PITG_08944* codes for a putative endoglucanase protein [15,17,18]. For gene *23123* no polymorphic sequences were identified in any isolate. Genetic analyses showed sequence variability for the other genes analyzed (*08944, 12737, 17063, 06099, 15980*), corresponding to synonymous and non-synonymous amino acid changes at the putative protein sequences. DNA sequences exhibited a wide range of values for the genetic parameters calculated for the genes analyzed, suggesting different evolutionary rates, mechanisms and/or selection pressures for each gene tested. Amino acid substitutions may have different effects on the corresponding protein depending on the position where it localizes [27]. Non-synonymous substitutions within the signal peptide as identified for putative proteins 08944 (L11S) and 17063 (I9P; I9S; S10P) may affect secretion. Gene 15980 is a putative non-secreted glycoside hydrolase; hence, mutations in the amino terminal region do not have an effect on secretion. Substitutions close to the RxLR or DEER motifs as found for 12737 (R57E) may affect the ability for protein translocation into the host cytoplasm in *P. infestans* [16]. The same situation was reported for the *P. falciparum* host targeting signal, where sequences surrounding the core RxLXE/D/Q were found to also contribute to the translocation of virulence proteins into the host erythrocytes [33]. Amino acid changes localized in the C-terminal region were found for putative proteins 06099, 08944, 12737, 15980 and 17063. As little as one or two amino acid variations in this region may cause changes in the virulence/avirulence patterns, as has been demonstrated for the *Avr3* effector gene of *P. infestans* where just two amino acid changes in positions 80 and 103 (KI/EM, respectively) are responsible for the change in avirulence/virulence phenotype [16]. In the present work, amino acid substitutions were localized in all these three regions. However, our current knowledge of pathogen and hosts studied here does not allow us to determine if amino acid substitutions identified in effector sequences are responsible for changes in avirulence/virulence phenotypes. Therefore, further functional research is needed to elucidate if these changes affect the avirulence/virulence characteristics of effector proteins in the different hosts. This analysis is fundamental for a plant breeding program because it allows the selection of those plant genotypes harboring corresponding R resistance proteins against prevalent races or variants of *P. infestans* in a given population.

Some genes are known and well characterized avirulent/virulent alleles in *P. infestans* populations [15,16,18,21,34]. The RxLR class of genes encode for most avirulent proteins known today from *P. infestans* such as *Avr2*, *Avr3a*, *Avr4*, *Avr*-*blb1*, *Avr*-*blb2* and others [17,25,35,36]. Here, we sequenced three genes coding for the RxLR class of putative effector proteins *17063*, *06099* and *12737*, for which corresponding R proteins are not known. Sequence polymorphisms, gene expression suppression or gene deletion are responsible for evasion of the plant recognition by corresponding plant resistance proteins [16,34,35]. These mechanisms are possible because of the functional redundancy present in the *P. infestans* effector repertoire, in which a gene may replace the function of another gene. Therefore, some research groups have focused on this group of proteins to identify host defense mechanisms looking for durable and stable resistance [20]. As gene *23123* did not show sequence variation in any isolate, it is a good candidate for further studies aimed to identify potential mechanisms to evade plant recognition such as gene loss, copy number variation or change in expression patterns that may hamper plant defense activation. In the same way, it is important to identify and clone the corresponding *R* resistance gene to test its capacity of inducing durable late blight resistance in crops. Proteins 06099 and 17063 from *P. infestans* induced cell death in the solanaceous non-host pepper (*Capsicum* sp.). This result may be useful for the identification of non-host resistance determinants or R proteins in pepper that eventually can be deployed in *P. infestans* hosts conferring late blight disease resistance. Extreme variability rates, recombination and diversifying selection have been reported for virulence proteins of *P. infestans* and other oomycete plant pathogens such as scr74, ATR1^NdWsB^ and ATR13 [10,11,12]. Non-synonymous amino acid substitutions such as those observed for the putative RxLR proteins 12737, 17063 and 06099 tested in our work may have been driven by co-evolution with a host factor as demonstrated for the *Avr3a*-*R3a* and *Avr2*-*R2* genes by gene interactions in *P. infestans* populations [16,37]. This knowledge has already been used to rapidly identify and clone *R* genes that were used in plant breeding programs [4,20,21]. Thereby, identification and cloning corresponding R genes will increase the available sources of resistance to the late blight disease that may be deployed by breeding programs in commercial varieties of crop plants.

In our research, different statistics were applied for identification of selection pressures that produced results exhibiting variable levels of significance. Several works have been performed to compare the different methods of selection identification finding contrasting results, which is in agreement with our findings [38,39,40,41]. Genes *08944* and *12737* were found under significantly negative selection in the present work, indicating that these genes are potentially under active evolution. Gene *12737* codes for a RxLR effector protein, and gene *08944* codes for a putative secreted endoglucanase protein [15,18]. In previous research, positive selection was found for more than two-thirds of the analyzed RxLR paralogues in other oomycete pathogens such as *P. sojae*, *P. ramorum* and *H. parasitica* [13]. More recently, negative selection has been reported for the *Avr3a* RxLR effector gene from *P. infestans* [19]. Interestingly, *Avr3a* gene was also found to be under negative selection by neutrality tests in a recent work performed in Colombian populations of *P. infestans* sensu lato [28]. Avr3a protein is recognized by the plant R protein R3a, triggering a hypersensitive response. R3a was introgressed in commercial varieties from *S. demissum* during the twentieth century, which were rapidly defeated by *P. infestans*. It is tempting to speculate that negative selection against the allelic variant of Avr3a recognized by R3a (i.e., Avr3a^KI^), in favor of the allelic variant not recognized (i.e., Avr3a^EM^), may confer an advantage to *P. infestans* strains for evasion of recognition by the corresponding R3a resistance protein present in potato varieties [16,19].

Endoglucanase proteins such as the *P. infestans* secreted effector 08944 belong to the glycosyl hydrolase 12 (cellulase H) family, which are involved in polysaccharide catabolic processes needed for plant cell wall degradation. Such pathogen effectors are important for tissue penetration, and plants evolved proteins that may recognize them or their degradation products known as DAMPs (damage associated molecular patterns) triggering defense responses. Putative protein 08944 exhibited several amino acid substitutions in an isolate of the *13*_*A2* lineage of *P. infestans* populations in Great Britain [18]. The *P. infestans 13*_*A2* lineage is highly aggressive on cultivated potatoes and displaced other *P. infestans* lineages in Great Britain in less than three years. Genomics of a *13*_*A2* isolate revealed extensive non-synonymous mutations in effector genes including *08944*, which showed several unique single-nucleotide polymorphic (SNP) sites likely contributing to observed virulence and aggressiveness of this isolate [18]. Therefore, it is expected that selection pressure was exerted at some point during the effector coevolution of genes *12737* and *08944* with corresponding plant *R* resistance genes, supporting significant results of the Tajima’s D test applied. Negative selection has also been reported in other effector genes of plant pathogens such as *Pseudomonas syringae* [42], *Microbotryum lychnidis*-*dioicae* [43], *Xanthomonas campestris* pv. vesicatoria [44], *Cladosporium fulvum* [27] and *M. oryzae* [45]. In addition, genomic analysis of effector genes in the *13*_*A2* isolate allowed the identification of potential targets for deploying resistance in crop varieties exemplifying the importance of the study of sequences of effector genes [18].

In some plant pathogen interactions, the evolutionary selection is exerted on a few and precise amino acid positions that are involved in protein–protein interactions as has been exemplified by the Avr3a protein of *P. infestans*. Phylogenetic analyses of effector genes in the present research are consistent with non-synonymous mutations at specific points over which selection pressures are eventually exerted. Such a pattern explains the low diversity in both nucleotide and amino acid sequences observed between isolates. Most branches grouped in phylogenetic trees correspond to the different haplotypes of amino acid sequences identified in the protein sequence alignments (Supplementary Figures S1–S5). Haplotypes were not related to geographic place or host of origin, hence indicating that more research is fundamental to fully understand the significance of each amino acid substitution.

Protein variability and gene duplication are important mechanisms for adaptation of pathogen populations to challenging environments such as a new host with new resistance mechanisms or agronomical practices [46]. Diversity in pathogenicity-related proteins has been observed for most groups of plant pathogens, including bacteria, virus, fungi and oomycetes [27,46,47]. DNA polymorphisms identified in the avirulence genes from *Cladosporium fulvum* are either non-synonymous modifications or complete gene deletions, or both, that are generally associated with transitions from avirulence to virulence [27]. Genetic diversity is usually higher in natural populations of the host plants and pathogens, as evidenced by the avirulence gene *ATR1^NdWsB^* from *H. parasitica* that showed extreme levels of polymorphism when sequenced from natural populations of *A. thaliana* [12]. Commercial varieties present limited genetic variability and new or specific variants of pathogen strains that can overcome plant recognition receptors or suppress plant immunity, adapt rapidly to crop plants defeating resistance and usually inducing devastating epidemics. Most studies of *P. infestans* populations have been made on crop fields. For a better understanding of effector gene diversity, it will be important to include more geographical regions with contrasting edaphoclimatic conditions, different cultivated and wild hosts and a high number of isolates. Further research is needed to confirm that the genes *12737* and *08944* found under selection in the present work are actually coevolving with host factors. It will be very important to identify and analyze interacting proteins from the host. Effector biology studies, named effectoromics, have emerged as a discipline to accelerate discovery and characterization of plant resistance genes, with proven utility for plant breeding programs around the world.

More studies to evaluate if there are allelic variants that can be recognized or evade recognition by the Andean Solanaceous hosts in Colombia would be a complement to the present work. It would be crucial to determine if isolates from wild hosts may infect crop hosts and to study the potential implications of results. As evolutionary selection was identified for two genes analyzed (08944, 12737), studies to determine what are the main effects of that selection in the recognition by host R genes would be very important, especially under the Andean conditions in Colombia where the crops are cultivated all year round, and highly favorable conditions for *P. infestans* development are present in places where crops are cultivated and cohabit with wild hosts.

## 4. Materials and Methods

### 4.1. Isolates

Plant samples were collected from several wild and cultivated hosts in different geographic regions from Colombia, South America. Twenty isolates were collected in previous surveys and maintained at Corpoica La Selva (Rionegro, Antioquia, Colombia) in liquid nitrogen [23]. Nine isolates were collected during the present research (Table 1).

Plant tissues showing typical late blight lesions, preferably with profuse sporulation, were collected, put into humid paper towels, packed in paper bags, kept in containers and sent to the laboratory of Fitotecnia Tropical at Universidad Nacional de Colombia sede Medellín for further analysis. Collection points were georeferenced using a GPS (Trimble^®^), and information about host, crop management, locality and altitude was registered where available. Plant tissues were rinsed in tap water and washed in phosphate-free soap with neutral pH (10% in sterile distilled water (SDW); Protokimica, Medellín, Colombia), then rinsed in SDW, dried in paper towels at room temperature and incubated in high-humidity chambers (>90%) at 16–18 °C until abundant sporulation was observed (usually between 3 to 5 days depending on the host). The tissues profusely sporulated were rinsed with SDW and sporangia recovered by filtration (10 µm mesh). Once sporangia were collected, the resulting suspensions were filtered (0.10 µm, Millipore™) to eliminate contaminating bacteria and fungi. Sporangia were re-suspended in SDW, and the concentration was adjusted to 1 × 10^4^ sporangia mL^−1^ using a Neubauer chamber. A total of 100 µL of the sporangia suspension was kept at −20 °C for further DNA purification. Remaining suspensions with sporangia from each isolate were inoculated into potato (*S. tuberosum* L. subsp. *andigena* Hawkes) tuber slices of cv. Tuquerreña, Careta, Sabanera, Tuquerreña or Nevada or another R gene-free variety susceptible to the late blight disease [48]. Inoculated potato slices were incubated in a growing chamber (Sanyo) in darkness at 16–18 °C until superficial mycelia and profuse sporulation were observed (usually about 5–7 days) [48].

Samples collected from tree tomato (*Solanum betaceaum* Cav.) showing lesions with profuse sporulation were processed in a similar way, but due to the difficulty that was exhibited to grow potato slices, they were grown in fully expanded leaves of tree tomato of 4 months old. If inocula were in high concentration (>1 × 10^4^ sporangia/mL), sporangia were transferred to semi-synthetic Rye-agar media (60 g of rye flour, 18 g of sucrose, 20 g of bacto-agar, 25 mg/L of β-sitosterol and SDW up to one liter) [48]; or if low concentrations were observed, sporangia were re-inoculated into potato slices or tree tomato leaves to obtain a high enough concentration of sporangia to be transferred to Rye-agar media [48].

For isolate purification in semi-selective media (Rye A, or tree tomato media), plant tissues with necrotic spots were washed in a solution of sodium hypochlorite (1%) in SDW for 1 min, followed by immersion in SDW for 1 min, then immersion in ethanol (70%) in SDW for 30 s and a final rinse in SDW for 30 s. Pieces of about 2 mm^2^ were cut from the edge of the lesion including green tissue and were placed in Petri dishes containing solid Rye A or tree tomato agar media (125 g of tree tomato pulp (*Solanum betaceum*). Samples were blended in 500 mL of SDW, filtered through four layers of cheesecloth, and 250 mL of obtained suspension was mixed with 100 mL of green pea broth (75 g boiled for 30 min in 300 mL of water). Samples were then combined with CaCO_3_ (0.5 g/L), sucrose (18 g/L) and bacteriological agar (20 g/L), the pH adjusted to 6.2 with NaOH 1N [28,49] and supplemented with antibiotics and fungicides (mycostatin 50 mg/L; benomyl 50 mg/L; chloramphenicol 50 mg/L; rifampicin (50 mg/L), kanamycin (25 mg/L) and ampicillin (50 mg/L)). Petri dishes were incubated at 16 °C in darkness until typical *P. infestans* colonies with sporangia were visible in the stereomicroscope (Nikon, Tokyo, Japan), and morphology was confirmed under light microscopy (Nikon Ni, coupled with DIC system, Tokyo, Japan). Purified isolates were sub-cultured every month in semi-selective media and incubated at 16–18 °C without antibiotics or fungicides.

For cryopreservation, sporangia were recovered as described before. The concentration was adjusted to 5 × 10^4^ sporangia mL^−1^, DMSO was added to a final concentration of 15%, cryovials were slowly and gently frozen in a cry-cool tank until they reached −40 °C, and then they were transferred and kept in liquid nitrogen until use. For isolate recovery from liquid nitrogen storage, frozen sporangia suspensions were thawed at room temperature, and suspensions were grown on potato slices as described. Once sporulation was observed, sporangia were filtered and concentration adjusted as described [48].

### 4.2. DNA Purification

To produce mycelia for nucleic acid extraction, isolates were grown in pea broth (120 g of fresh or frozen pea, SDW up to 1 L of media broth) [48] at 20 °C for about 2 weeks until abundant mycelia were obtained. Mycelia were filtered through filter paper (Whatman No 1, Buckinghamshire, UK) and then were freeze-dried for 12 h and kept at −80 °C until further use. Freeze-dried *P. infestans* mycelia were ground in liquid nitrogen to a fine powder, and DNA was purified using the DNeasy^®^ plant mini kit (Qiagen^®^, Hilden, Germany) or the Norgen Plant/fungi DNA isolation kit following the manufacturer’s instructions. Purified DNA was eluted in nuclease-free SDW or Tris HCl 10 mM pH 8.0 (50–100 μL). Quality and quantity of DNA were analyzed by agarose gel electrophoresis (1% in 1 × TBE buffer pH 8.0 (0.09 M Tris-Borate, 0.002 M EDTA)) at 100 V for 1 h, stained with SYBR Safe™ DNA gel stain (Invitrogen™, Molecular probes™, Waltham, USA) (5 μL of dye per 100 mL 1 × TBE buffer) and concentration measured by a Nanodrop^®^ ND-1000 spectrophotometer (Wilmington, USA). Purified DNA was kept at −20 °C until further use.

### 4.3. Mating Type

Purified DNA and primers S1A (5′-AGGATTTCAACAA) and S1B (5′-TGCTTCCTAAGG) were used for the polymerase chain reaction (PCR) amplification of a DNA fragment of about 1250 bp linked to the S1 locus, which is associated with the A1 mating type [50,51]. The following components were added to a PCR tube of 200µL (UltraAmp PCR products, Sorenson Biosciences, Salt Lake City, UT, USA): 2.5 µL of buffer 10× for *Taq* polymerase TopTaq^®^ (Tris·Cl, KCl, (NH_4_)_2_SO_4_, 15 mM MgCl_2_, pH 8.7, Qiagen); 2.5 µL of CoralLoad™ 10× (Qiagen, Hilden, Germany); 5 µL of Q-solution™ 5× (Qiagen, Hilden, Germany); 0.5 µL of dNTP 2.5 mM; 0.5 µL of forward primer 10 µM; 0.5 µL of reverse primer 10 µM; 0.125 µL of *Taq* DNA polymerase (TopTaq, Qiagen, Hilden, Germany); 1 µL of DNA template 200 ng/µL; and molecular biology grade water to a final volume of 25 µL (usually 17.375 µL).

PCR reaction mix was gently homogenized, briefly spun down and processed on thermal cycler equipment (LabNet International Inc, model MultiGene OptiMax, Edison, USA) under the following program: initial denaturation at 94 °C for 30 s, followed by 35 cycles of amplification each consisting of initial denaturation at 94 °C for 30 s, 35 °C for 30 s and 72 °C for 30 s. After the 35 cycles, a final extension at 72 °C for 3 min was performed [51]. PCR products were analyzed by agarose gel electrophoresis (1.8%) in TBE 0.5× (Amresco, Solon, OH, USA), stained and visualized as described.

### 4.4. Effector Genes

Effector genes analyzed in the present work were selected from previous reports [15,18,25,52]. Full open reading frame (ORF) gene sequences of each gene were obtained [52]. Primers were designed for each gene using the software Primer3web following the default parameters and optimized for an annealing temperature of 60 °C (http://bioinfo.ut.ee/primer3/, 18 July 2017, Table 6) [53,54].

### 4.5. PCR Amplification and Sequence of Effector Genes

ORF of each selected gene from each isolate was amplified using specific primers. The following reaction components were added to a PCR tube of 200 µL (UltraAmp PCR products, Sorenson Biosciences, Salt Lake City, USA): 2.5 µL of buffer 10× for *Taq* polymerase TopTaq^®^ (Tris·Cl, KCl, (NH_4_)_2_SO_4_, 15 mM MgCl_2_, pH 8.7, Qiagen, Hilden, Germany); 2.5 µL of CoralLoad™ 10X (Qiagen, Hilden, Germany); 5 µL of Q-solution™ 5× (Qiagen, Hilden, Germany); 0.5 µL of dNTP 2.5 mM; 0.5 µL of primer forward 10 µM; 0.5 µL of primer reverse 10 µM; 0.125 µL of *Taq* DNA polymerase (TopTaq, Qiagen, Hilden, Germany); 1 µL of DNA template 200 ng/µL; and molecular biology grade water to a final volume of 25 µL (usually 17.375 µL).

PCR reaction was gently homogenized, briefly spun down and was put on thermal cycler equipment (LabNet International Inc, modelo MultiGene OptiMax, Edison, USA) under the following program: initial denaturation at 94 °C for 2 min, followed by 40 cycles of amplification each consisting of initial denaturation at 94 °C for 30 s, 60 °C for 60 s and 72 °C for 60 s. After the 35 cycles, a final extension at 72 °C for 10 min was performed. For each pair of primers, a negative control without DNA template was included. PCR products were analyzed by agarose (1.8%) gel electrophoresis in TBE 0.5× pH 8.0 (Amresco, Solon, OH, USA), stained and visualized as described. Three reactions were performed for each gene and for each isolate. PCR products showing a clear single amplification band of expected size were purified using the PCR QIAquick (Qiagen, Hilden, Germany) following the manufacturer’s instructions. Purified fragments were analyzed by agarose gel electrophoresis and quantified by a Nanodrop^®^ ND-1000 spectrophotometer (Wilmington, USA) as described. Purified PCR products were sent to the Macrogen (Seoul, Republic of Korea) capillary sequencing service following the company´s guidelines (http://foreign.macrogen.com/eng/business/seq_Standard%20Sequencing.html, accessed on 18 July 2017).

### 4.6. Sequence Analyses

Gene sequences were manually assembled, cleaned and edited using Bioedit© software version 7.2.6.1 (http://www.mbio.ncsu.edu/BioEdit/bioedit.html, 18 July 2017) [55,56]. All sequences were compared to the corresponding sequence in the genome of isolate T30–4 available on the NCBI webpage (https://www.ncbi.nlm.nih.gov/bioproject/17665, 19 July 2017) [52]. Sequences were converted to Fasta format, and the full ORF sequence set of each gene was aligned and polymorphic sites identified using the Clustal W algorithm [57] implemented in Bioedit software. DNA sequences obtained were registered in GenBank under accession numbers MH429133-MH429161 (Gene *PITG 06099*, gi|301113030); MH429162-MH429190 (Gene *PITG 08944*, gi|301107979); MH429191- MH429217 (Gene *PITG 12737*, gi|301102350); MH429218-MH429245 (Gene *PITG 17063*, ID:XM_002897216.1); MH429246-MH429257 (Gene *PITG 15980*, gi|301097656); and MH429258-MH429286 (Gene *PITG 23123*, gi|301093980) (Table S1). Putative protein sequences were identified using the ExPASy software. Genetic and phylogenetic analyses were performed using DnaSP v. 5.10.01 and MEGAX software [58,59,60].

The Maximum Likelihood method was used for construction of the phylogenetic tree of each gene. The best substitution model was selected according to the Bayesian information criteria (BIC) using the lowest value. Bootstrap analysis with 1000 iterations was performed. Bootstrap values below 50 were not shown in figures. For other variables, the default parameters in MEGAX for phylogenetic reconstruction were used [58,61,62].

For each gene, the codon number, number of variable sites (S), total number of mutations (ETA), mutation rate of the population theta (per site) (θ), number of haplotypes (H), haplotype (gene) diversity (Hd), variance of haplotype diversity, standard deviation of haplotype diversity and nucleotide diversity (per site) (pi) were calculated. Neutrality tests were calculated using the Fu and Li´s F, Fu and Li´s D, Fu’s Fs, Strobeck’s S and Tajima´s D statistics to identify whether the non-synonymous over synonymous substitution rate values (dN/dS) found were significant.

## Figures and Tables

**Table 1:** Host and geographic location of isolates collected.

Isolate Code/Number	Municipality/Department	Host	Meters Above the Sea Level (masl)	Geographic Coordinates	Physiological Race	Virulence Factors	Reference
M1	La Ceja/Antioquia	*Solanum quitoense* cv. Castilla	2152	NA	NA	NA	Collected in the present work
M-1	La Ceja/Antioquia	*Solanum betaceum* cv. Común	2152	LT 5.940655, LG 75.427367	NA	NA	Collected in the present work
M-2	La Ceja/Antioquia	*Solanum* sp.	2176	LT 5.946915, LG 75.422601	NA	NA	Collected in the present work
M-3	La Ceja/Antioquia	*Solanum* sp.	2184	LT. 5.969591, LG. 75.492130	NA	NA	Collected in the present work
M-7	La Ceja/Antioquia	*Solanum quitoense* cv. Castilla	2473	LT. 5.962374, LG. 75.453905	NA	NA	Collected in the present work
M-8	La Ceja/Antioquia	*Solanum* sp	2390	NA	NA	NA	Collected in the present work
M-9	La Ceja/Antioquia	*Solanum betaceum* cv. Común	2410	LT. 5.982741, LG. 75444036	NA	NA	Collected in the present work
M-3	El Peñol/Antioquia	*Solanum lycopersicum* cv. Chonto	1976	NA	NA	NA	Collected in the present work
M-10	Entrerríos/Antioquia	*Solanum* sp.	2575	NA	NA	NA	Collected in the present work
OP-1	Oporapa/Huila	*Solanum quitoense* cv. Castilla	NA	NA	4.11	2	[23]
OP-2	Oporapa/Huila	*Solanum quitoense* cv. Castilla	NA	NA	1.4.8.11	2	[23]
OP-3	Oporapa/Huila	*Solanum quitoense* cv. Castilla	NA	NA	4.8	2	[23]
OP-5	Oporapa/Huila	*Solanum quitoense* cv. Castilla	NA	NA	4.8	2	[23]
UR-1	Urrao/Antioquia	*Solanum quitoense* cv. Sin espinas	NA	NA	8	1	[23]
UR-5	Urrao/Antioquia	*Solanum quitoense* cv. Castilla	NA	NA	0.4.8.11	4	[23]
UR-9	Urrao/Antioquia	*Solanum quitoense* cv. Castilla	NA	NA	0.4.8.11	4	[23]
UR-18	Urrao/Antioquia	*Solanum quitoense* cv. Castilla	NA	NA	0.4.8	3	[23]
UR-24	Urrao/Antioquia	*Solanum quitoense* cv. Sin espinas	NA	NA	0.4.8.11	4	[23]
JA-4	Jardín/Antioquia	*Solanum quitoense* cv. Castilla	NA	NA	4.8.11	3	[23]
JA-5	Jardín/Antioquia	*Solanum quitoense* cv. Castilla	NA	NA	4.8.10.11	4	[23]
JA-6	Jardín/Antioquia	*Solanum quitoense* cv. Castilla	NA	NA	4.8	2	[23]
GP-3	Guatapé/Antioquia	*Solanum quitoense* cv. Castilla	NA	NA	4.8	2	[23]
ST-1	Santuario/Antioquia	*Solanum quitoense* cv. Castilla	NA	NA	4.8	2	[23]
ST-4	Santuario/Antioquia	*Solanum quitoense* cv. Castilla	NA	NA	3.4.8	3	[23]
GA-1	Garzón/Huila	*Solanum quitoense* cv. Castilla	NA	NA	4.8	2	[23]
MB-1	Montebello/Antioquia	*Solanum quitoense* cv. Castilla	NA	NA	4.8.11	3	[23]
VE-1	Versalles/Valle	*Solanum quitoense* cv. Castilla	NA	NA	NA	NA	[23]
SELVA-2	Rionegro/Antioquia	*Solanum quitoense* cv. Castilla	NA	NA	0.4	2	[23]
SR-2	Santa Rosa/Risaralda	*Solanum quitoense* cv. Castilla	NA	NA	4.7.8.11	4	[23]

NA: not available.

**Table 2:** Genetic analyses of effector genes from *Phytophthora infestans* isolates.

Gene Number	Codon Number	Number of Variable Segregating Sites (Polymorphic) (S)	Total Number of Mutations (Eta)	Number of Haplotypes (h)	Haplotype Diversity (gene) (Hd)	Variance of Haplotype Diversity	Standard Deviation of Haplotype Diversity
*08944*	240	52	53	5	0.253	0.01076	0.104
*12737*	168	16	16	5	0.270	0.01188	0.109
*17063*	160	6	7	4	0.315	0.01165	0.108
*06099*	143	6	7	8	0.692	0.00557	0.075
*15980*	443	3	3	3	0.641	0.00936	0.097
*23123* *	-	-	-	-	-	-	-

* No polymorphisms were identified for gene *23123*.

**Table 3:** Genetic analysis of effector genes from *Phytophthora infestans*.

GENE	Nucleotide Diversity (Per Site) (Pi)	Sampling Variance of Pi	Standard Deviation of Pi	Average Number of Nucleotide Differences (k)	Theta Per Sequence	Theta Per Site	Number of Non-Synonymous Substitutions
*08944*	0.00488	0.0000123	0.00351	3.53103	13.37825 from Eta	0.01850 from Eta	21
*12737*	0.00363	0.0000033	0.00182	1.83862	4.11157 from S, Theta-W	0.00811 from S, Theta-W	10
*17063*	0.00188	0.0000005	0.00071	0.90640	1.78245 from Eta	0.00370 from Eta	2
*06099*	0.00294	0.0000003	0.00055	1.26882	1.75220 from Eta	0.00406 from Eta	7
*15980*	0.00116	0.0000000	0.00015	1.53846	0.96674 from S, Theta-W	0.00073 from S, Theta-W	2
*23123* *	-	-	-	-	-	-	-

* No polymorphisms were identified for gene *23123*.

**Table 4:** Amino acid substitutions in the putative effector proteins.

Gene	Polymorphic Site (aa)	Amino Acid in Genome Sequence T30-4 *	Substitutions
*06099*	39	Q	P
	63	S	RR
	80	E	Q, K
	153	K	R
*08944*	11	L	S
	160	G	C
	173	V	X
	182	X	C
	185	E	Q
	190	V	I
	192	G	D
	202	P	S
	203	gap	E
	206	H	Y
	207	V	D
	208	A	V
	209	P	S
	210	D	Y
	212	I	P
	217	G	T
	219	R	W
	220	H	S
	221	G	P
	222	A	T
	223	F	I
	224	P	L
	228	W	R
	229	gap	R
	230	D	H
	231	D	K
	232	C	F
*12737*	57	R	E
	77	R	I
	80	V	L
	94	V	G
	95	G	C, V
	99	M	L
	121	Y	F
	127	E	Q
	147	G	V
	150	D	N
*15980*	303	F	Y
	309	G	R
*17063*	9	I	P, S
	10	S	P
	99	Q	L

* All sequences were compared to the corresponding sequence in the genome of isolate T30–4 available on the NCBI webpage (https://www.ncbi.nlm.nih.gov/bioproject/17665, 19 July 2017).

**Table 5:** Neutrality tests for non-synonymous and synonymous substitutions.

GENE	Tajima’s D	Fu and Li’s D	Fu and Li’s F	Fu’s Fs	Strobeck’s S
***08944***	−2.75121. Statistical significance: ***, *p* **< 0.001**	−5.19552. Statistical significance: **, *p* **< 0.02**	−5.18444. Statistical significance: **, *p* **< 0.02**	3.551	0.083. (Probability that NHap <= 5). Probability that [NHap = 5]: 0.056
***12737***	−1.90412. Statistical significance: *, *p* < 0.05	−2.09581. Statistical significance: Not significant, 0.10 > *p* > 0.05	−2.38643. Statistical significance: Not significant, 0.10 > *p* > 0.05	0.962	0.490. (Probability that NHap <= 5). Probability that [NHap = 5]: 0.214
*17063*	−1.46408 Statistical significance: Not significant, *p* > 0.10	−0.10632 Statistical significance: Not significant, *p* > 0.10	−0.59832 Statistical significance: Not significant, *p* > 0.10	0.133	0.716. (Probability that NHap <= 4). Probability that [NHap = 4]: 0.249
*06099*	−0.80876. Statistical significance: Not significant, *p* > 0.10	−0.83518. Statistical significance: Not significant, *p* > 0.10	−0.96515. Statistical significance: Not significant, *p* > 0.10	−2.900	0.983. (Probability that NHap <= 8). Probability that [NHap = 8]: 0.035
*15980*	1.86987. Statistical significance: Not significant, 0.10 > *p* > 0.05	1.08633. Statistical significance: Not significant, *p* > 0.10	1.45939. Statistical significance: Not significant, 0.10 > *p* > 0.05	1.752	0.392. (Probability that NHap <= 3). Probability that [NHap = 3]: 0.244
*23123* *	-	-	-	-	-

*No polymorphisms were identified for gene *23123*. Values were calculated using the total number of mutations.

**Table 6:** Primers designed to amplify the full open reading frame (ORF) of each gene analyzed.

GENE (PITG/Genbank Code Number)	GENE SIZE (bp)		PRIMER SEQUENCE
*06099*, gi|301113030, XM_002998240.1_T30-4_(*PITG_06099*)	689	FW* RV**	ATCTGGCCAGCCATTTGGAA GAACGCAAATGCTAATGACATGGA
*08944*, gi|301107979, XM_002902941.1_T30-4_(*PITG_08944*)	683	FW RV	TCGGCAATCTGCTTCAAGACAC TTGGCGGACTCTTGCATGTC
*12737*, gi|301102350, XM_002900083.1 T30-4 (*PITG_12737*)	707	FW RV	TTTTCTCGTCCAACGCCACA TCGACATCGCCCACAATTTC
*15980*, gi|301097656, XM_002897722.1 T30-4 (*PITG_15980*)	1532	FW RV	GCCACCCAGTAGATTCGCTCA CGCAAGCACGTCCAGCTCTA
*17063*, XM_002897216.1 T30-4 (*PITG_17063*)	683	FW RV	CGCGCATCAGAAGGTGTTTG CACCGCCCGAAGCAAATTTAT
*23123*, gi|301093980, XM_002997715.1 T30-4 SCR50 (*PITG_23123*)	353	FW RV	CACCGCAACAACCGAGTCAC AGGACGGATGTGGGGAATCA

*FW: primer forward; **RV: primer reverse.

## Supplementary Materials

The following are available online at https://www.mdpi.com/2076-0817/9/7/551/s1, **Figure S1.** Alignment of putative amino acid sequence of protein coded by effector gene PITG_06099, **Figure S2.** Alignment of putative amino acid sequence of protein coded by effector gene PITG_08944, **Figure S3.** Alignment of putative amino acid sequence of protein coded by effector gene PITG_12737, **Figure S4.** Alignment of putative amino acid sequence of protein coded by effector gene PITG_15980, **Figure S5.** Alignment of putative amino acid sequence of protein coded by effector gene PITG_17063, **Figure S6.** Molecular Phylogenetic analysis by Maximum Likelihood method of gene *06099*, **Figure S7.** Molecular Phylogenetic analysis by Maximum Likelihood method of gene *12737*, **Figure S8.** Molecular Phylogenetic analysis by Maximum Likelihood method of gene *15980*, **Figure S9.** Molecular Phylogenetic analysis by Maximum Likelihood method of gene *17063*, **Figure S10.** Molecular Phylogenetic analysis by Maximum Likelihood method of gene *08944*, **Table S1.** The full list of accession numbers registered at Genbank.
