# Supplementary material for: Allelic Variation and Selection in Effector Genes of Phytophthora infestans (Mont.) de Bary
Source: Pathogens. 2020 Jul 9;9(7):551. doi: 10.3390/pathogens9070551 (PMC7400436; doi:10.3390/pathogens9070551)
Supplement: Supplementary file 1 [file pathogens-09-00551-s001.pdf]

Pathogens **2020**, *9*, 551; doi:10.3390/pathogens9070551

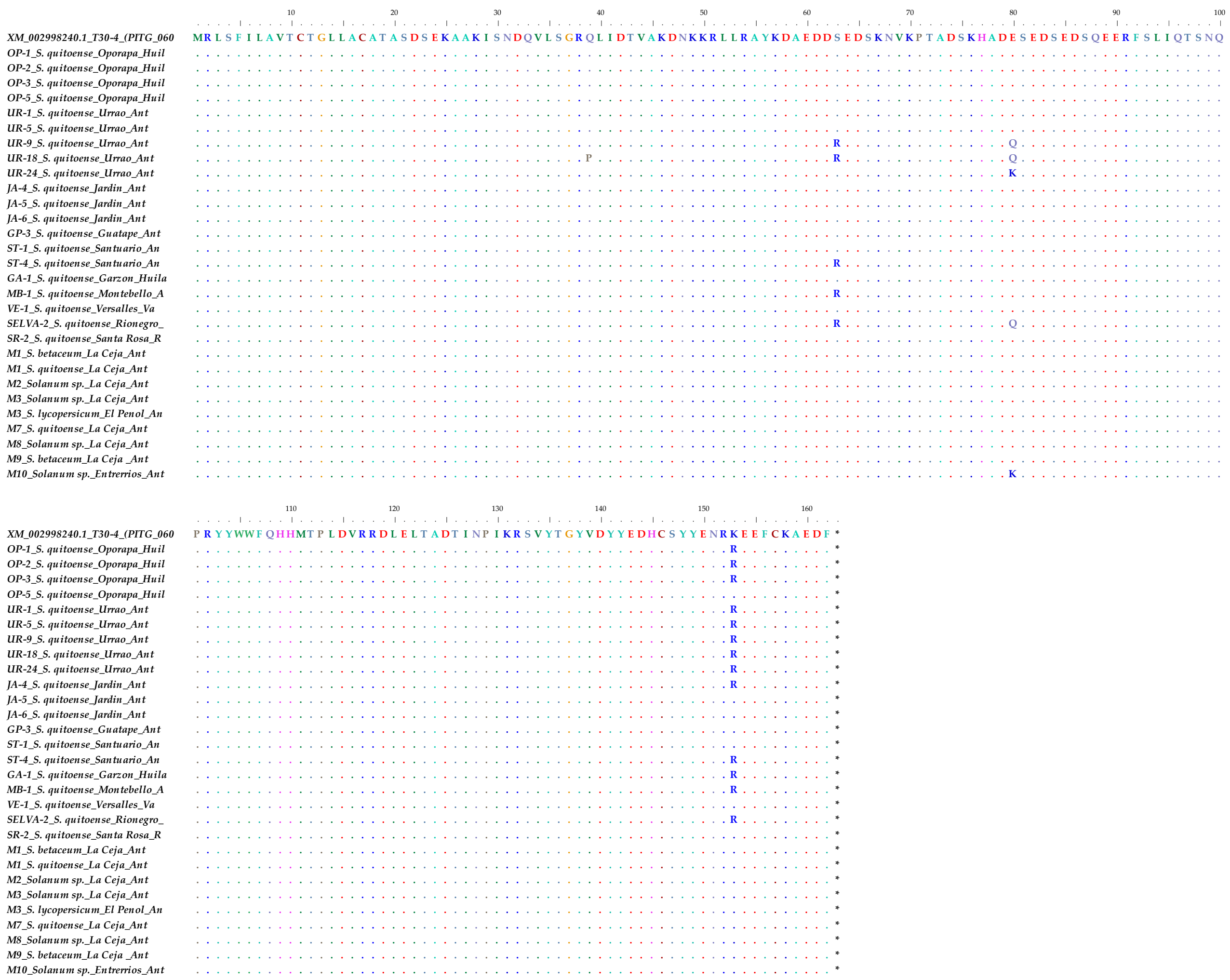

Figure S1. Alignment of putative amino acid sequence of protein coded by effector gene PITG_06099. Dots represent sequence positions without amino acid substitutions when compared to the reference sequence of genome T-30.

Pathogens **2020**, *9*, 551; doi:10.3390/pathogens9070551

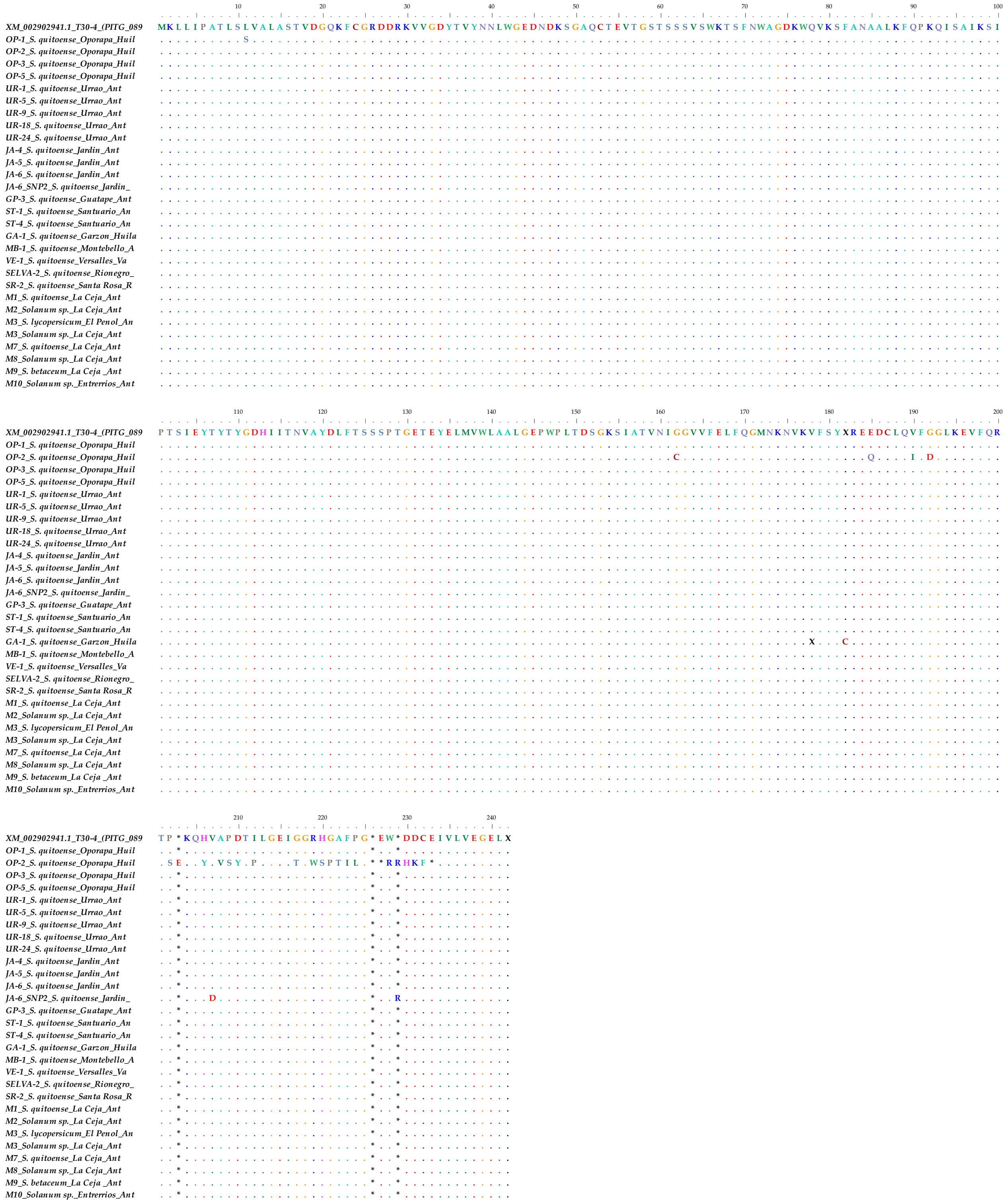

Figure S2. Alignment of putative amino acid sequence of protein coded by effector gene PITG_08944. Dots represent sequence positions without amino acid substitutions when compared to the reference sequence of genome T-30.

Pathogens **2020**, *9*, 551; doi:10.3390/pathogens9070551

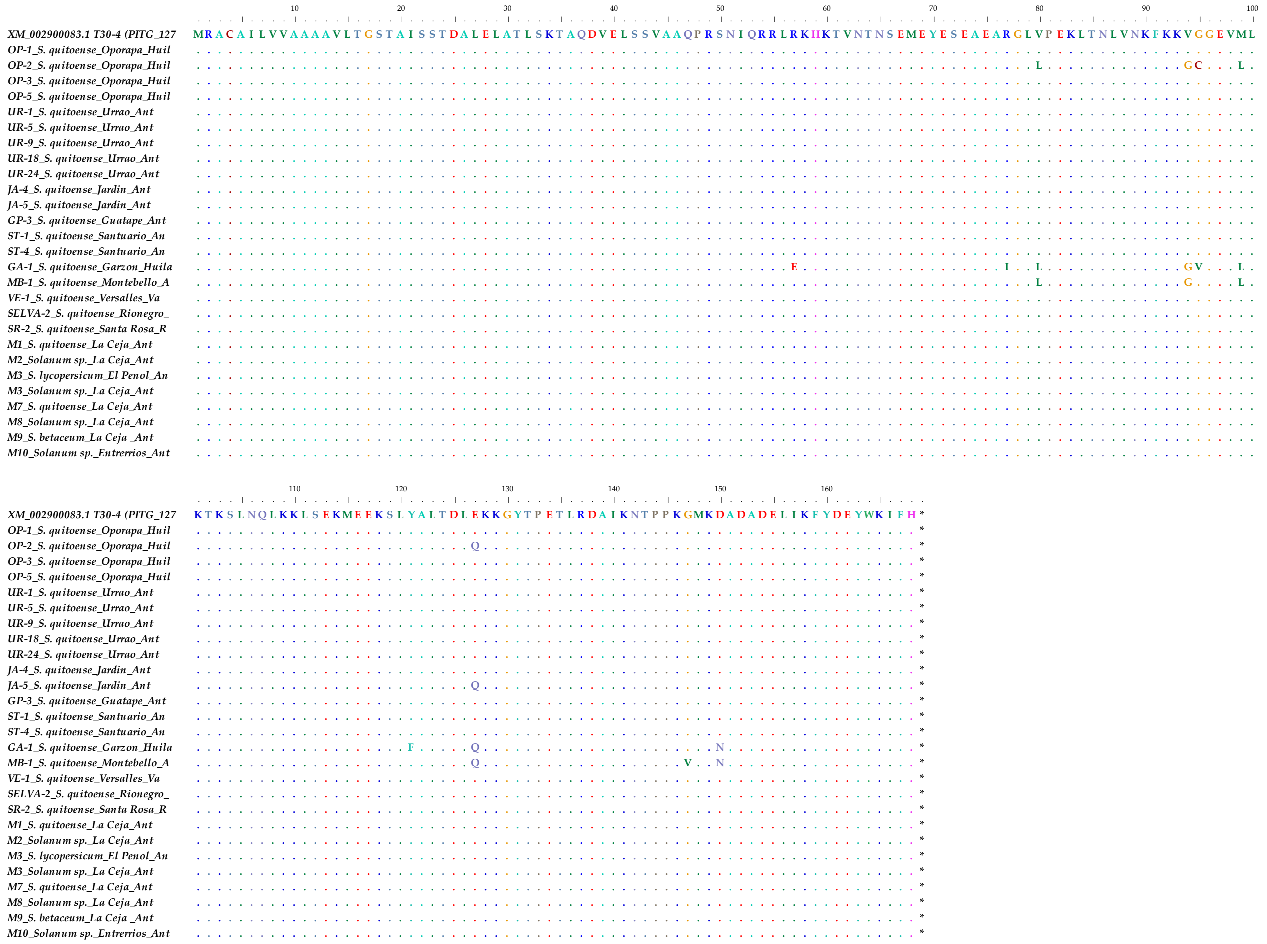

Figure S3. Alignment of putative amino acid sequence of protein coded by effector gene PITG_12737. Dots represent sequence positions without amino acid substitutions when compared to the reference sequence of genome T-30.

Pathogens **2020**, *9*, 551; doi:10.3390/pathogens9070551

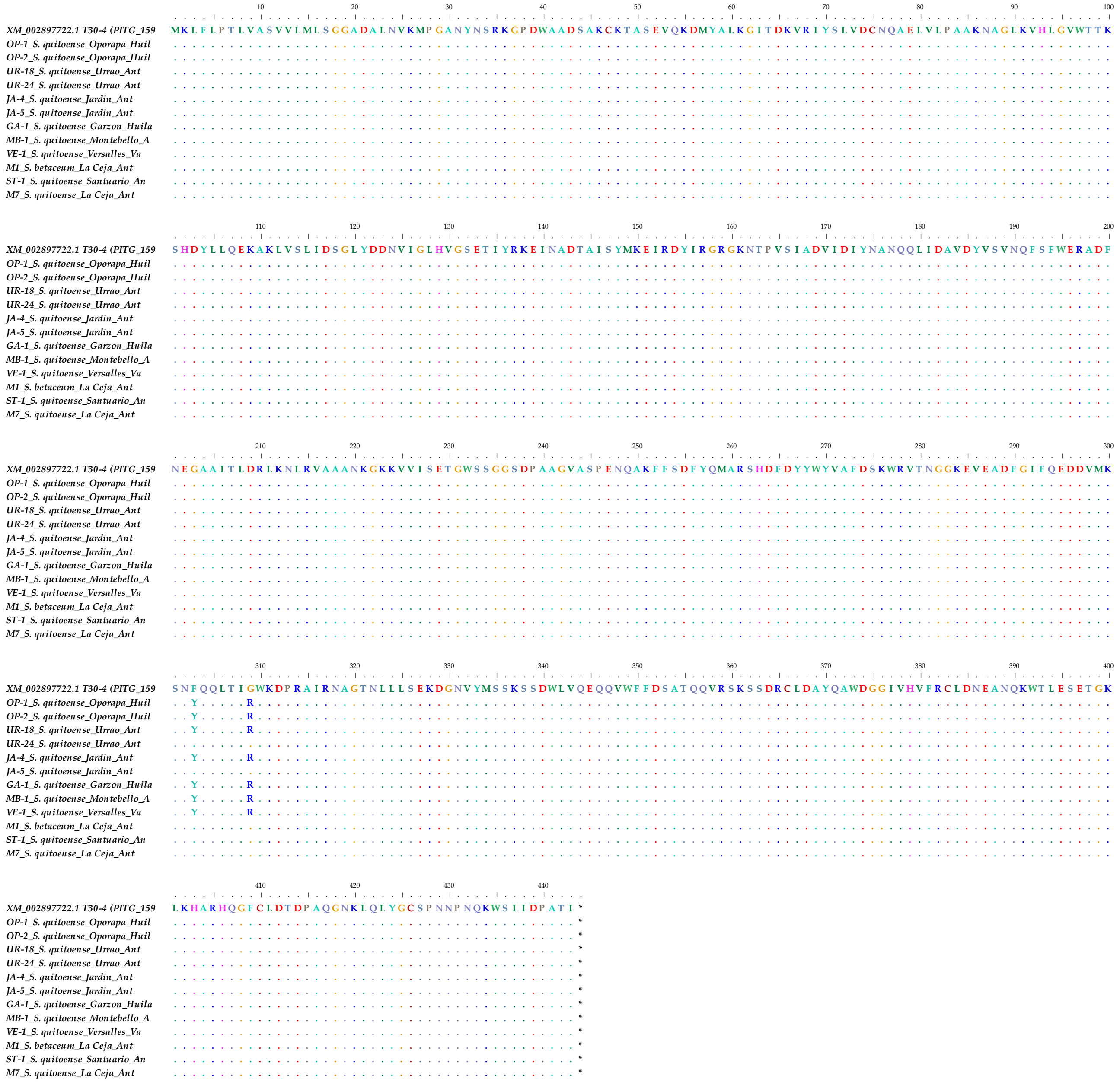

Figure S4. Alignment of putative amino acid sequence of protein coded by effector gene PITG_15980. Dots represent sequence positions without amino acid substitutions when compared to the reference sequence of genome T-30.

Pathogens **2020**, *9*, 551; doi:10.3390/pathogens9070551

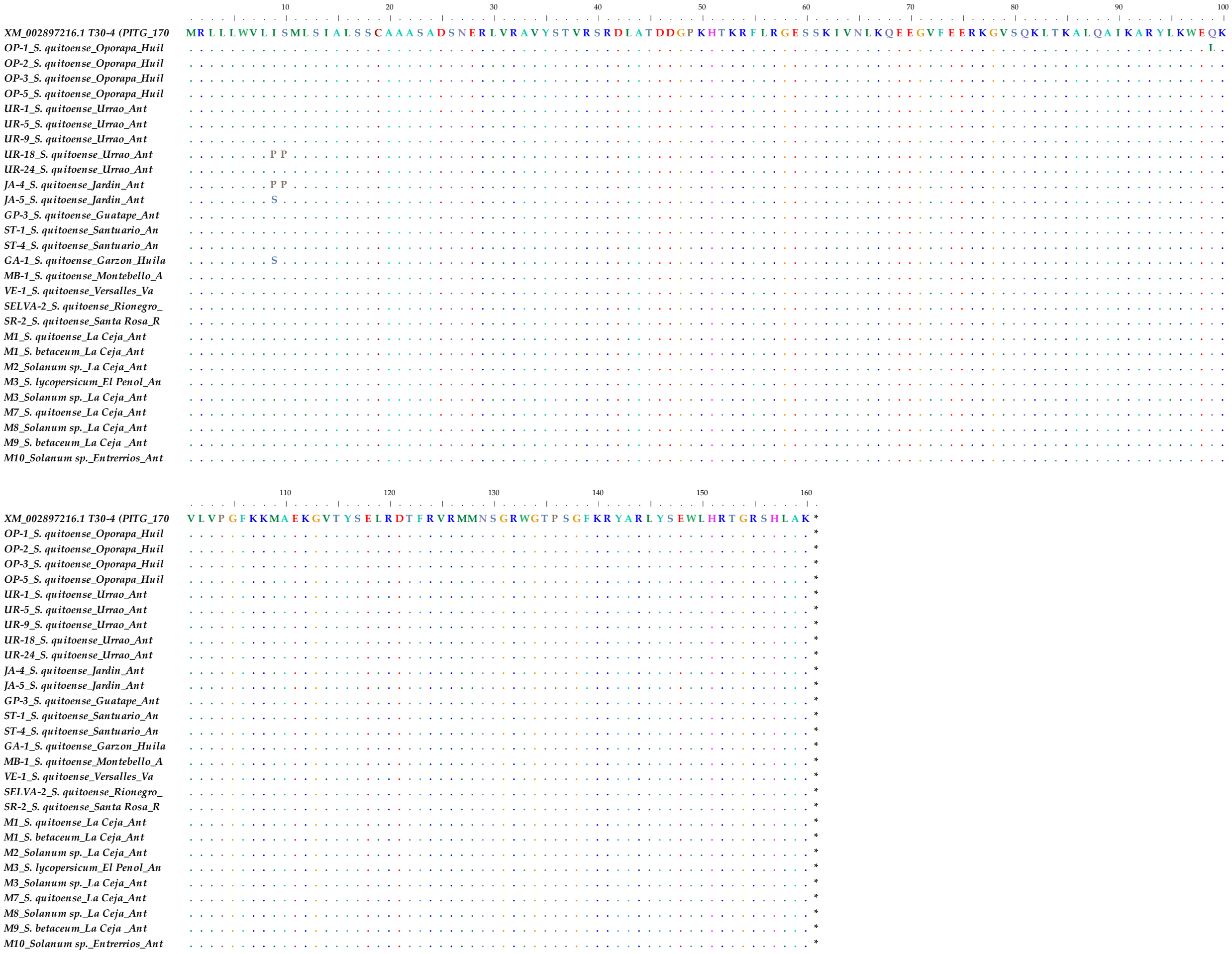

Figure S5. Alignment of putative amino acid sequence of protein coded by effector gene PITG_17063. Dots represent sequence positions without amino acid substitutions when compared to the reference sequence of genome T-30.

Pathogens **2020**, *9*, 551; doi:10.3390/pathogens9070551

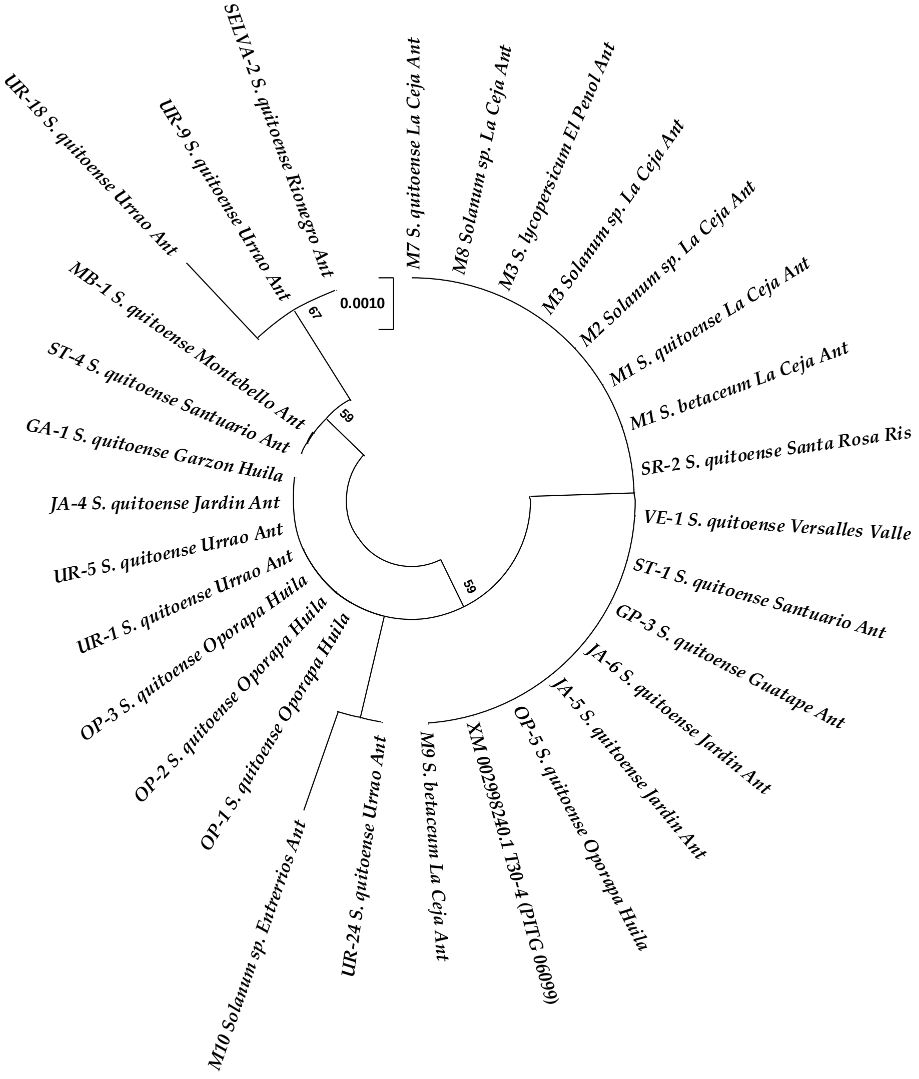

Figure S6. Molecular Phylogenetic analysis by Maximum Likelihood method of gene 06099.
The evolutionary history was inferred by using the Maximum Likelihood method and Jukes-Cantor model. The tree with the highest log likelihood (-727.66) is shown. The percentage of trees in which the associated taxa clustered together is shown next to the branches. Initial tree(s) for the heuristic search were obtained automatically by applying Neighbor-Join and BioNJ algorithms to a matrix of pairwise distances estimated using the Jukes-Cantor model, and then selecting the topology with superior log likelihood value. The tree is drawn to scale, with branch lengths measured in the number of substitutions per site. This analysis involved 30 nucleotide sequences. Codon positions included were 1st+2nd+3rd+Noncoding. There were a total of 489 positions in the final dataset. The bootstrap consensus tree was inferred from 1000 replicates. Branches corresponding to partitions reproduced in less than 50% bootstrap replicates were collapsed. Labels are organized as follows: first, isolate code in Table 1; second, host scientific name (e.g., *Solanum quitoense*, *S. betaceum*, *S. lycopersicum*, *Solanum* sp.); third, Municipality and Department of Colombia from where corresponding isolate was collected (e.g., La Ceja Ant). Ant: Department of Antioquia, Colombia. Valle: Department of Valle del Cauca, Colombia. Ris: Department of Risaralda, Colombia. Evolutionary analyses were conducted in MEGA X [58].

Pathogens **2020**, *9*, 551; doi:10.3390/pathogens9070551

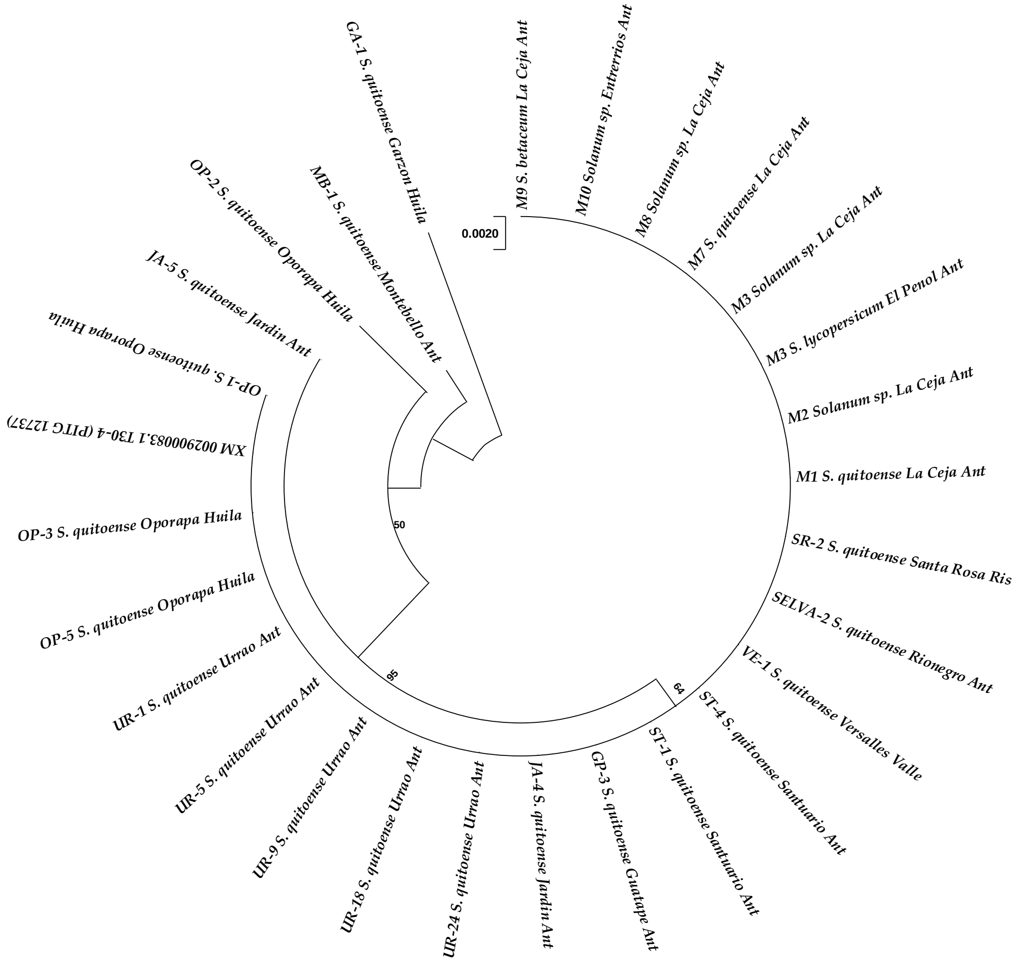

Figure S7. Molecular Phylogenetic analysis by Maximum Likelihood method of gene 12737.
The evolutionary history was inferred by using the Maximum Likelihood method and Jukes-Cantor model. The tree with the highest log likelihood (-820.95) is shown. The percentage of trees in which the associated taxa clustered together is shown next to the branches. Initial tree(s) for the heuristic search were obtained automatically by applying Neighbor-Join and BioNJ algorithms to a matrix of pairwise distances estimated using the Jukes-Cantor model, and then selecting the topology with superior log likelihood value. The tree is drawn to scale, with branch lengths measured in the number of substitutions per site. This analysis involved 28 nucleotide sequences. Codon positions included were 1st+2nd+3rd+Noncoding. There were a total of 507 positions in the final dataset. The bootstrap consensus tree was inferred from 1000 replicates. Branches corresponding to partitions reproduced in less than 50% bootstrap replicates were collapsed. Labels are organized as follows: first, isolate code in Table 1; second, host scientific name (e.g., *Solanum quitoense*, *S. betaceum*, *S. lycopersicum*, *Solanum* sp.); third, Municipality and Department of Colombia from where corresponding isolate was collected (e.g., La Ceja Ant). Ant: Department of Antioquia, Colombia. Valle: Department of Valle del Cauca, Colombia. Ris: Department of Risaralda, Colombia. Evolutionary analyses were conducted in MEGA X [58]

Pathogens **2020**, *9*, 551; doi:10.3390/pathogens9070551

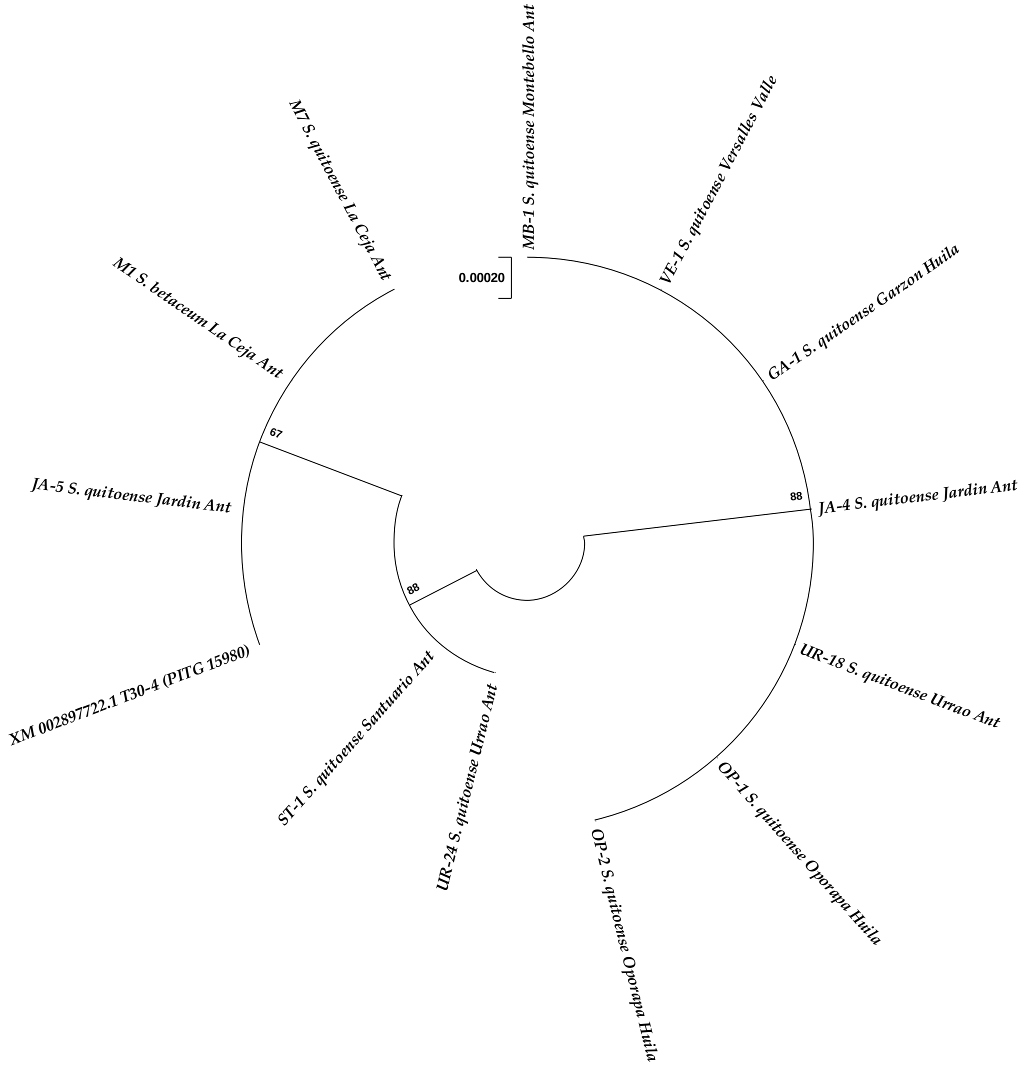

Figure S8. Molecular Phylogenetic analysis by Maximum Likelihood method of gene 15980.
The evolutionary history was inferred by using the Maximum Likelihood method and Jukes-Cantor model. The tree with the highest log likelihood (-1873.04) is shown. The percentage of trees in which the associated taxa clustered together is shown next to the branches. Initial tree(s) for the heuristic search were obtained automatically by applying Neighbor-Join and BioNJ algorithms to a matrix of pairwise distances estimated using the Jukes-Cantor model, and then selecting the topology with superior log likelihood value. The tree is drawn to scale, with branch lengths measured in the number of substitutions per site. This analysis involved 13 nucleotide sequences. Codon positions included were 1st+2nd+3rd+Noncoding. There were a total of 1332 positions in the final dataset. The bootstrap consensus tree was inferred from 1000 replicates. Branches corresponding to partitions reproduced in less than 50% bootstrap replicates were collapsed. Labels are organized as follows: first, isolate code in Table 1; second, host scientific name (e.g., *Solanum quitoense*, *S. betaceum*, *S. lycopersicum*, *Solanum* sp.); third, Municipality and Department of Colombia from where corresponding isolate was collected (e.g., La Ceja Ant). Ant: Department of Antioquia, Colombia. Valle: Department of Valle del Cauca, Colombia. Ris: Department of Risaralda, Colombia. Evolutionary analyses were conducted in MEGA X [58]

Pathogens **2020**, *9*, 551; doi:10.3390/pathogens9070551

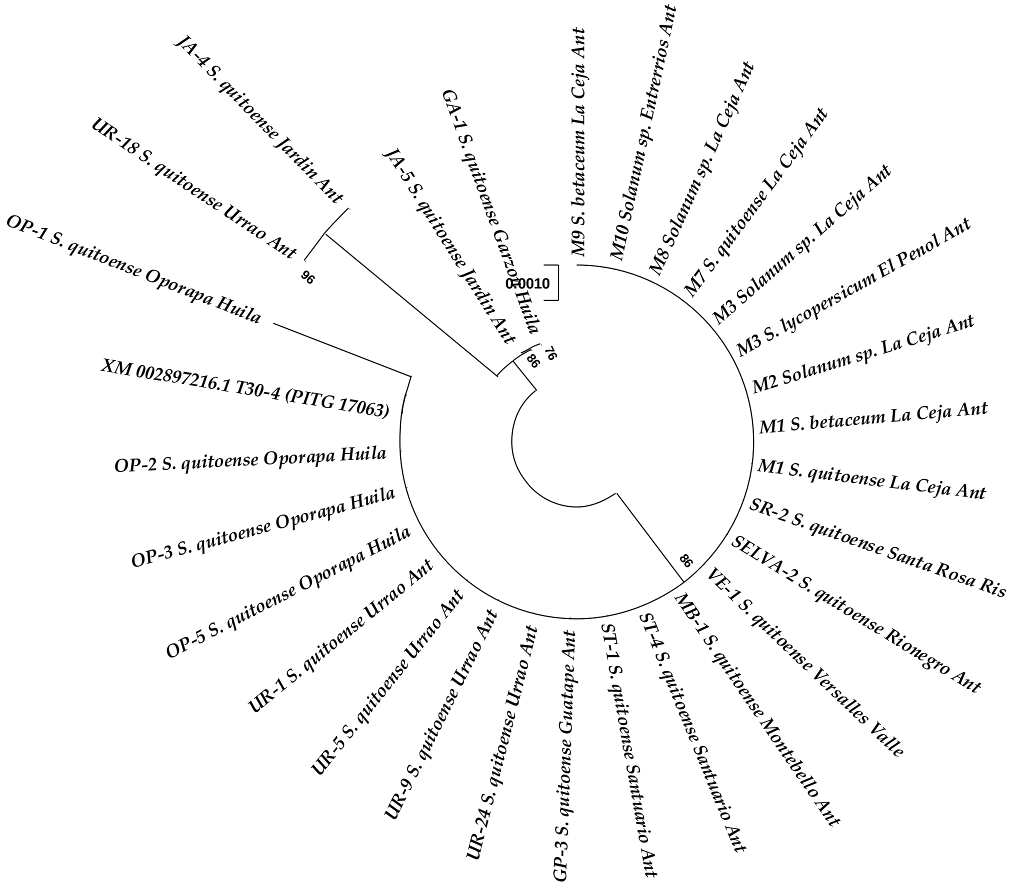

Figure S9. Molecular Phylogenetic analysis by Maximum Likelihood method of gene 17063.
The evolutionary history was inferred by using the Maximum Likelihood method and Jukes-Cantor model. The tree with the highest log likelihood (-721.46) is shown. The percentage of trees in which the associated taxa clustered together is shown next to the branches. Initial tree(s) for the heuristic search were obtained automatically by applying Neighbor-Join and BioNJ algorithms to a matrix of pairwise distances estimated using the Jukes-Cantor model, and then selecting the topology with superior log likelihood value. The tree is drawn to scale, with branch lengths measured in the number of substitutions per site. This analysis involved 29 nucleotide sequences. Codon positions included were 1st+2nd+3rd+Noncoding. There were a total of 483 positions in the final dataset. The bootstrap consensus tree was inferred from 1000 replicates. Branches corresponding to partitions reproduced in less than 50% bootstrap replicates were collapsed. Labels are organized as follows: first, isolate code in Table 1; second, host scientific name (e.g., *Solanum quitoense*, *S. betaceum*, *S. lycopersicum*, *Solanum* sp.); third, Municipality and Department of Colombia from where corresponding isolate was collected (e.g., La Ceja Ant). Ant: Department of Antioquia, Colombia. Valle: Department of Valle del Cauca, Colombia. Ris: Department of Risaralda, Colombia. Evolutionary analyses were conducted in MEGA X [58]

Pathogens **2020**, *9*, 551; doi:10.3390/pathogens9070551

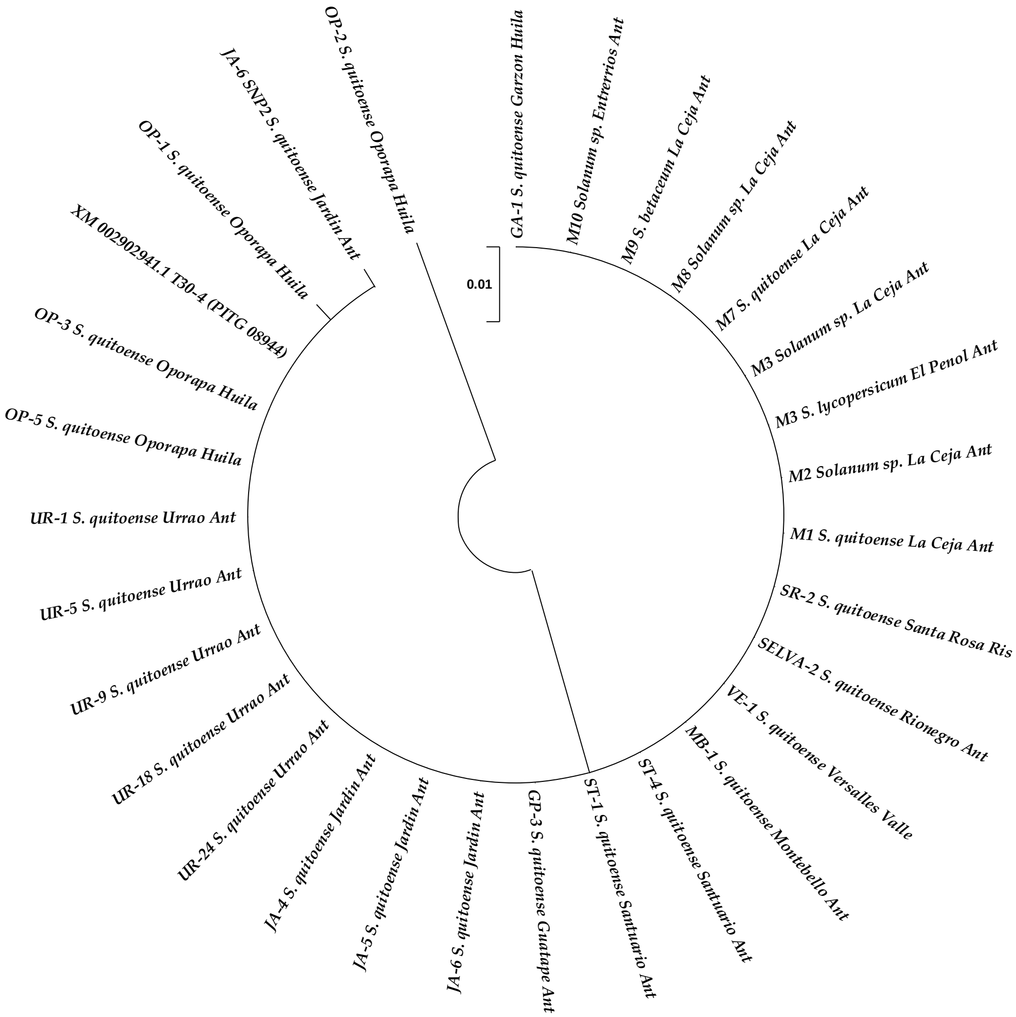

Figure S10. Molecular Phylogenetic analysis by Maximum Likelihood method of gene 08944.
The evolutionary history was inferred by using the Maximum Likelihood method and Jukes-Cantor model. The tree with the highest log likelihood (-1238.15) is shown. The percentage of trees in which the associated taxa clustered together is shown next to the branches. Initial tree(s) for the heuristic search were obtained automatically by applying Neighbor-Join and BioNJ algorithms to a matrix of pairwise distances estimated using the Jukes-Cantor model, and then selecting the topology with superior log likelihood value. The tree is drawn to scale, with branch lengths measured in the number of substitutions per site. This analysis involved 30 nucleotide sequences. Codon positions included were 1st+2nd+3rd+Noncoding. There were a total of 724 positions in the final dataset. The bootstrap consensus tree was inferred from 1000 replicates. Branches corresponding to partitions reproduced in less than 50% bootstrap replicates were collapsed. Labels are organized as follows: first, isolate code in Table 1; second, host scientific name (e.g., *Solanum quitoense*, *S. betaceum*, *S. lycopersicum*, *Solanum* sp.); third, Municipality and Department of Colombia from where corresponding isolate was collected (e.g., La Ceja Ant). Ant: Department of Antioquia, Colombia. Valle: Department of Valle del Cauca, Colombia. Ris: Department of Risaralda, Colombia. Evolutionary analyses were conducted in MEGA X [58]

Table S1. The full list of accession numbers registered at Genbank

Pathogens **2020**, *9*, 551; doi:10.3390/pathogens9070551

BankIt2118581 1-OP-1_06099	MH429133
BankIt2118581 2-OP-2_06099	MH429134
BankIt2118581 3-OP-3_06099	MH429135
BankIt2118581 4-OP-5_06099	MH429136
BankIt2118581 5-UR-1_06099	MH429137
BankIt2118581 6-UR-5_06099	MH429138
BankIt2118581 7-UR-9_06099	MH429139
BankIt2118581 8-UR-18_06099	MH429140
BankIt2118581 9-UR-24_06099	MH429141
BankIt2118581 17-JA-4_06099	MH429142
BankIt2118581 18-JA-5_06099	MH429143
BankIt2118581 JA-6_3_06099	MH429144
BankIt2118581 15-GP-3_06099	MH429145

Pathogens **2020**, *9*, 551; doi:10.3390/pathogens9070551

BankIt2118581 10-ST-1_06099	MH429146
BankIt2118581 11-ST-4_06099	MH429147
BankIt2118581 14-GA-1_06099	MH429148
BankIt2118581 16-MB-1_06099	MH429149
BankIt2118581 19-VE-1_06099	MH429150
BankIt2118581 13-SELVA_06099	MH429151
BankIt2118581 12-SR-2_06099	MH429152
BankIt2118581 M1_TARBOL_3_06099	MH429153
BankIt2118581 M1_LULO_3_06099	MH429154
BankIt2118581 M2_SSP_3_06099	MH429155
BankIt2118581 M3_SSP_3_06099	MH429156
BankIt2118581 M3_SLYC_3_06099	MH429157
BankIt2118581 M7_LULO_3_06099	MH429158

Pathogens **2020**, *9*, 551; doi:10.3390/pathogens9070551

BankIt2118581 M8_SSP_3_06099	MH429159
BankIt2118581 M9_TARBOL_3_06099	MH429160
BankIt2118581 M10SSP_3_06099	MH429161
BankIt2118747 AG82_O8944_OP-1	MH429162
BankIt2118747 AG84_O8944_OP-2	MH429163
BankIt2118747 OP-3_1_08944	MH429164
BankIt2118747 OP-5_1_08944	MH429165
BankIt2118747 UR-1_1_08944	MH429166
BankIt2118747 UR-5_1_08944	MH429167
BankIt2118747 AG85_O8944_UR-9	MH429168
BankIt2118747 AG83_O8944_UR-18	MH429169
BankIt2118747 UR-24_1_08944	MH429170
BankIt2118747 AG86_O8944_JA-4	MH429171

Pathogens **2020**, *9*, 551; doi:10.3390/pathogens9070551

BankIt2118747 AG87_O8944_JA-5 MH429172

BankIt2118747 JA-6_1_08944 MH429173

BankIt2118747 JA-6_1_08944_2_SNP MH429174

BankIt2118747 GP-3_1_08944 MH429175

BankIt2118747 ST-1_1_08944 MH429176

BankIt2118747 ST-4_1_08944 MH429177

BankIt2118747 AG81_O8944_GA-1 MH429178

BankIt2118747 MB-1_1_08944 MH429179

BankIt2118747 AG88_O8944_VE-1 MH429180

BankIt2118747 SELVA-2_1_08944 MH429181

BankIt2118747 SR-2_1_08944 MH429182

BankIt2118747 M1-LULO_1_08944 MH429183

BankIt2118747 M2-SSP_1_08944 MH429184

Pathogens **2020**, *9*, 551; doi:10.3390/pathogens9070551

BankIt2118747 M3-SLYC_1_08944	MH429185
BankIt2118747 M3-SSP_1_08944	MH429186
BankIt2118747 M7-LULO_1_08944	MH429187
BankIt2118747 M8-SSP_1_08944	MH429188
BankIt2118747 M9-TARBOL_1_08944	MH429189
BankIt2118747 M10-SSP_1_08944	MH429190
BankIt2118749 AG130_12737_OP-1	MH429191
BankIt2118749 AG129_12737_OP-2	MH429192
BankIt2118749 OP-3_3_12737	MH429193
BankIt2118749 OP-5_3_12737	MH429194
BankIt2118749 UR-1_3_12737	MH429195
BankIt2118749 UR-5_3_12737	MH429196
BankIt2118749 AG131_12737_UR-9	MH429197

Pathogens **2020**, *9*, 551; doi:10.3390/pathogens9070551

BankIt2118749 UR-18_3_12737	MH429198
BankIt2118749 UR-24_3_12737	MH429199
BankIt2118749 JA-4_3_12737	MH429200
BankIt2118749 AG134_12737_JA-5	MH429201
BankIt2118749 GP-3_3_12737	MH429202
BankIt2118749 ST-1_3_12737	MH429203
BankIt2118749 ST-4_3_12737	MH429204
BankIt2118749 AG137_12737_GA-1	MH429205
BankIt2118749 AG135_12737_MB-1	MH429206
BankIt2118749 AG136_12737_VE-1	MH429207
BankIt2118749 SELVA-2_3_12737	MH429208
BankIt2118749 SR-2_3_12737	MH429209
BankIt2118749 M1-LULO_3_12737	MH429210

Pathogens **2020**, *9*, 551; doi:10.3390/pathogens9070551

BankIt2118749 M2-SSP_3_12737	MH429211
BankIt2118749 M3-SLYC_3_12737	MH429212
BankIt2118749 M3-SSP_3_12737	MH429213
BankIt2118749 M7-LULO_3_12737	MH429214
BankIt2118749 M8-SSP_3_12737	MH429215
BankIt2118749 M9-TARBOL_3_12737	MH429216
BankIt2118749 M10-SSP_3_12737	MH429217
BankIt2118754 AG118_17063_OP-1	MH429218
BankIt2118754 AG117_17063_OP-2	MH429219
BankIt2118754 OP-3_4_17063	MH429220
BankIt2118754 OP-5_4_17063	MH429221
BankIt2118754 UR-1_4_17063	MH429222
BankIt2118754 UR-5_4_17063	MH429223

Pathogens **2020**, *9*, 551; doi:10.3390/pathogens9070551

BankIt2118754 AG116_17063_UR-9	MH429224
BankIt2118754 AG115_17063_UR_18	MH429225
BankIt2118754 AG114_17063_UR-24	MH429226
BankIt2118754 AG113_17063_JA-4	MH429227
BankIt2118754 AG112_17063_JA-5	MH429228
BankIt2118754 GP-3_4_17063	MH429229
BankIt2118754 ST-1_4_17063	MH429230
BankIt2118754 ST-4_4_17063	MH429231
BankIt2118754 AG109_17063_GA-1	MH429232
BankIt2118754 AG111_17063_MB-1	MH429233
BankIt2118754 AG110_17063_VE-1	MH429234
BankIt2118754 SELVA-2_4_17063	MH429235
BankIt2118754 SR-2_4_17063	MH429236

Pathogens **2020**, *9*, 551; doi:10.3390/pathogens9070551

BankIt2118754 M1-LULO_4_17063	MH429237
BankIt2118754 M1-TARBOL_4_17063	MH429238
BankIt2118754 M2-SSP_4_17063	MH429239
BankIt2118754 M3-SLYC_4_17063	MH429240
BankIt2118754 M3-SSP_4_17063	MH429241
BankIt2118754 M7-LULO_4_17063	MH429242
BankIt2118754 M8-SSP_4_17063	MH429243
BankIt2118754 M9-TARBOL_4_17063	MH429244
BankIt2118754 M10-SSP_4_17063	MH429245
BankIt2118755 AG128_15980_OP-1	MH429246
BankIt2118755 AG127_15980_OP-2	MH429247
BankIt2118755 AG125_15980_UR-18	MH429248
BankIt2118755 AG124_15980_UR-24	MH429249

Pathogens **2020**, *9*, 551; doi:10.3390/pathogens9070551

BankIt2118755 AG123_15980_JA-4 MH429250

BankIt2118755 AG122_15980_JA-5 MH429251

BankIt2118755 AG119_15980_GA-1 MH429252

BankIt2118755 AG121_15980_MB-1 MH429253

BankIt2118755 AG120_15980_VE-1 MH429254

BankIt2118755 M1-TARBOL_2_15980 MH429255

BankIt2118755 ST-1_2_15980 MH429256

BankIt2118755 M7-LULO_2_15980 MH429257

BankIt2118756 AG80_23123_(OP-1) MH429258

BankIt2118756 AG79_23123_(OP-2) MH429259

BankIt2118756 OP-3_2_23123 MH429260

BankIt2118756 OP-5_2_23123 MH429261

BankIt2118756 UR-1_2_23123 MH429262

Pathogens **2020**, *9*, 551; doi:10.3390/pathogens9070551

BankIt2118756 UR-5_2_23123	MH429263
BankIt2118756 AG78_23123_(UR-9)	MH429264
BankIt2118756 AG77_23123_(UR-18)	MH429265
BankIt2118756 AG76_23123_(UR-24)	MH429266
BankIt2118756 AG75_23123_(JA-4)	MH429267
BankIt2118756 AG74_23123_(JA-5)	MH429268
BankIt2118756 JA-6_2_23123	MH429269
BankIt2118756 GP-3_2_23123	MH429270
BankIt2118756 ST-1_2_23123	MH429271
BankIt2118756 ST-4_2_23123	MH429272
BankIt2118756 AG71_23123_(GA-1)	MH429273
BankIt2118756 AG73_23123_(MB-1)	MH429274
BankIt2118756 AG72_23123_(VE-1)	MH429275

Pathogens **2020**, *9*, 551; doi:10.3390/pathogens9070551

BankIt2118756 SELVA-2_2_23123	MH429276
BankIt2118756 SR-2_2_23123	MH429277
BankIt2118756 M1_TARBOL_2_23123	MH429278
BankIt2118756 M1_LULO_2_23123	MH429279
BankIt2118756 M2_SSP2_23123	MH429280
BankIt2118756 M3_SSP_2_23123	MH429281
BankIt2118756 M3_SLYC_2_23123	MH429282
BankIt2118756 M7_LULO_2_23123	MH429283
BankIt2118756 M8_SSP_2_23123	MH429284
BankIt2118756 M9_TARBOL_2_23123	MH429285
BankIt2118756 M10_SSP_2_23123	MH429286
